# Hypotheses in light detection by vertebrate ancient opsin in the bird brain

**DOI:** 10.1111/jne.70020

**Published:** 2025-03-16

**Authors:** Tyler J. Stevenson, Timothy A. Liddle, Simone L. Meddle, Jonathan H. Pérez, Stuart N. Peirson, Russell G. Foster, Gaurav Majumdar

**Affiliations:** ^1^ School of Biodiversity, One Health and Veterinary Medicine University of Glasgow Glasgow UK; ^2^ The Roslin Institute The University of Edinburgh Edinburgh UK; ^3^ Department of Biology University of South Alabama Mobile Alabama USA; ^4^ Sleep and Circadian Neuroscience Institute University of Oxford Oxford UK; ^5^ Department of Zoology University of Allahabad Prayagraj India

**Keywords:** mediobasal hypothalamus, neuroscience, photobiology, photoreceptor, preoptic area

## Abstract

Extra‐retinal photoreception is common across fish and avian species. In birds, the hypothalamus contains non‐visual photoreceptors that detect light and regulate multiple endocrine systems. To date, light‐dependent control of seasonal reproduction is one of the most well‐studied systems that require deep brain photoreception. However, the precise photoreceptor(s) that detect light and the neuroendocrine connection between opsin‐expressing cells and the gonadotropin‐releasing hormone‐1 (GnRH1) system remain poorly defined. In the past couple of decades, two opsin molecules have been proposed to link light detection with seasonal reproduction in birds: neuropsin (Opn5) and vertebrate ancient opsin (VA opsin). Only VA opsin is expressed in GnRH1 cells and has an absorption spectrum that matches the action spectrum of the avian photoperiodic reproductive response. This perspective describes how the annual change in daylength, referred to as photoperiod, regulates the neuroendocrine control of seasonal reproduction. The opsin genes are then outlined, and the cellular phototransduction cascade is described, highlighting the common feature of hyperpolarization in response to light stimulation. We then discuss the latest evidence using short‐hairpin RNA to temporarily knock down VA opsin and Opn5 on transcripts involved in the neuroendocrine regulation of reproduction. Based on emerging data, we outline three theoretical scenarios in which VA opsin might regulate GnRH1 synthesis and release in birds. The models proposed provide a series of testable hypotheses that can be used to improve our understanding of avian light detection by VA opsin or other opsin‐expressing cells in the brain.

## 1 INTRODUCTION

Photon detection in animals is evolutionarily ancient and is achieved by either ciliary or rhabdomeric light receptors. The ocellus, a sensory organ in ascidians (i.e., sea squirts) contains several ciliary photoreceptors that, when activated, result in a graded hyperpolarization response in the photoreceptor cells.^1^, ^2^ Ciliary photoreceptors are proposed to be the origin of light detection in vertebrate retinal pigment cells^3^ and have diversified into a range of opsin molecules essential for vision.^4^ Opsin proteins are generally associated with visual‐forming photoreceptors and are classified as either cones (i.e., Opn1) or rods (i.e., Opn2). Rods function for scotopic vision and exhibit high sensitivity to light stimulation as well as slow dark adaptation.^5^ Photoreception in retinal ganglion cells is also crucial for nonimage‐forming functions, such as the entrainment of mammalian circadian rhythms by melanopsin (Opn4^6^, ^7^, ^8^). However, for most non‐mammalian species, light can be detected and transduced in multiple non‐retinal tissues. The first tissue used to characterize the molecular structure of a ciliary photoreceptor subtype, pinopsin, was the pineal gland in domestic chickens (*Gallus domesticus*).^9^, ^10^ It is now well established that extra‐retinal photoreception is common across nonmammalian animals and that photoreceptors in the brain regulate a wide range of physiological functions.^11^, ^12^ This perspective covers the role of the ciliary photoreceptors in light detection and photoperiod time measurement in the avian brain. We will first describe the role of day length in regulating seasonal reproduction in birds and then define how gonadotropin‐releasing hormone‐1 (GnRH1) neurons control gonadal growth and involution. We also discuss the criteria for light detection by the avian brain and include the latest molecular and functional evidence to support the conjecture that vertebrate ancient opsin (VA opsin) is the predominant non‐visual photoreceptor for the photoperiodic control of avian seasonal reproduction. We conclude with a series of theoretical models, referred to as Hypotheses, that account for how VA opsin might link photoreception to the GnRH1 system in birds.

## 2 THE AVIAN PHOTOPERIODIC RESPONSE

The annual changes in day length, also referred to as photoperiod, are the primary environmental cue that most birds use to time seasonal reproduction and migration.^13^, ^14^ Across most avian species, the vernal increase in photoperiod stimulates reproductive development and maturation, leading to a physiological state commonly termed “photostimulated.”^15^ Photostimulated birds are characterized by the elevated production of circulating sex steroids (e.g., testosterone and estrogens), large‐sized gonads, and gametogenesis. Prolonged exposure to long photoperiod (i.e., >12 h of light) in many bird species leads to reproductive inhibition and the involution of reproductive organs, a physiological state called photorefractoriness.^16^ Two types of photorefractoriness have been characterized in birds and have been termed absolute and relative photorefractoriness. The two criteria used to differentiate the types of photorefractoriness are (1) spontaneous gonadal involution despite continued exposure to long photoperiod and (2) lengthening the photoperiod has no effect on the gonads once photorefractoriness is established.^17^ Birds that are absolute photorefractory adhere to both criteria. Birds that are relative photorefractory, such as the Japanese quail (*Coturnix japonica*), must experience a decline in photoperiod to demonstrate gonadal involution.^18^ Birds must experience short photoperiod days (i.e., <12 h of light) in order to develop a physiological sensitivity to long photoperiod, a state called “photosensitive.”^15^ In some avian species, such as the European starling (*Sturnus vulgaris*), exposure to only 10 short day lengths is sufficient to re‐sensitize the brain to long photoperiod, as indicated by an increase in GnRH1,^19^, ^20^ and the birds become physiologically responsive to light stimulation.^15^ In hens, the timing of the ovulatory cycle and egg laying is in part dependent on dark phase duration.^21^ GnRH1 cells in the preoptic area of the hypothalamus are a cellular correlate of the avian photoperiodic response.^22^ In birds that display absolute photorefractoriness, the number of GnRH1‐expressing cells in the preoptic area increases during photostimulation and then declines to nearly undetectable levels during the onset of photorefractoriness.^17^, ^23^ The development of a photosensitive state is characterized by the gradual increase in the amount of GnRH1.^19^, ^20^, ^23^ GnRH release into the hypophyseal portal system is also regulated by photoperiodic cues and is controlled by tanycytes originating in the ependymal layer of the third ventricle. In quail, short photoperiod is associated with extended tanycyte terminals, which prevents GnRH nerve endings from contacting the basal lamina membrane.^24^ Exposure to stimulatory long photoperiods induces a retraction in tanycytes and permits GnRH release stimulating gonadotropin secretion. Long photoperiods stimulate thyrotropin‐stimulating hormone subunit beta (*Tshβ*) expression in the pars tuberalis, which subsequently signals the synthesis of deiodinase type‐2 (*Dio2*) in tanycytes. Increased Dio2 synthesis facilitates the production of local triiodothyronine, which causes tanycytes to retract.^25^ Over the past few years, significant attention has been devoted to understanding the link between light detection and the GnRH1 system in birds.

Localized illumination studies, conducted in the 1930's demonstrated that birds possess photoreceptors located deep within the brain, which regulate both daily and seasonal reproductive responses to day length.^26^ Neural lesions,^27^ and immediate early gene studies^28^ then implicated the mediobasal hypothalamus as the key region for the photoperiodic regulation of avian seasonal reproduction. Work by Foster et al.^29^ illustrated that the avian photoperiod response in Japanese quail was triggered by light with a wavelength of approximately 492 nm. In quail, *Tshβ* a gene implicated in the photoperiodic response^30^ shows peak sensitivity to wavelength between 479 and 500 nm (Nakane et al., 2019) which is highly similar to the wavelength originally published by Foster et al.^29^ and corresponds to the absorption spectrum of VA opsin (*λ*_max_ 492).^31^ The family of ciliary photoreceptors includes several well‐known opsins including rhodopsin and VA opsin which have been classified as Opn2 based on sequence structure and use the 11‐*cis* isoform of retinaldehyde to function as a sensory photopigment.^11^ The initial hypothesis that was tested was that a rhodopsin‐like molecule was critical for light detection in the bird brain.^29^, ^32^, ^33^ Several other opsins such melanopsin (Opn4), and neuropsin (Opn5) were subsequently proposed to be involved in the photoperiodic response based primarily on neuroanatomical distribution within the avian brain.^34^, ^35^ Of the ciliary photoreceptors within the Opn2 family, only VA opsin is distributed throughout the preoptic area (POA) and mediobasal hypothalamus, but also located in many other hypothalamic regions in the domestic chicken and Japanese quail brain.^36^ Crucially, VA opsin‐expressing cells exhibit an absorption spectrum that closely matches the avian photoperiodic response in Japanese quail (490 nm,^37^). Histological studies have also established that VA opsin^36^ and rhodopsin‐like immunoreactivity^38^ are co‐localized with GnRH1.

## 3 LIGHT DETECTION AND THE CELLULAR BASIS OF PHOTORECEPTOR SIGNAL TRANSDUCTION

Opsins are a family of proteins, commonly referred to as photopigments, that capture photons and function in visual and non‐visual phototransduction.^39^ Phototransduction is the intracellular cascade that links the photopigment to changes in cell membrane—hyperpolarisation in the case of rods and cones, and depolarization in the case of melanopsin and invertebrate opsin‐expressing cells. The first opsin was sequenced in 1982^40^, ^41^ and subsequently, the first opsin protein was visualized using x‐ray crystallography.^42^ Over 1000 different opsins have subsequently been identified in the genomes of virtually all eukaryotic organisms from jellyfish to primates.^31^ As the focus of this review is on light detection for non‐visual functions, the mechanisms of visual opsins will not be considered further (see Reference 43 for a review).

**FIGURE 1:**
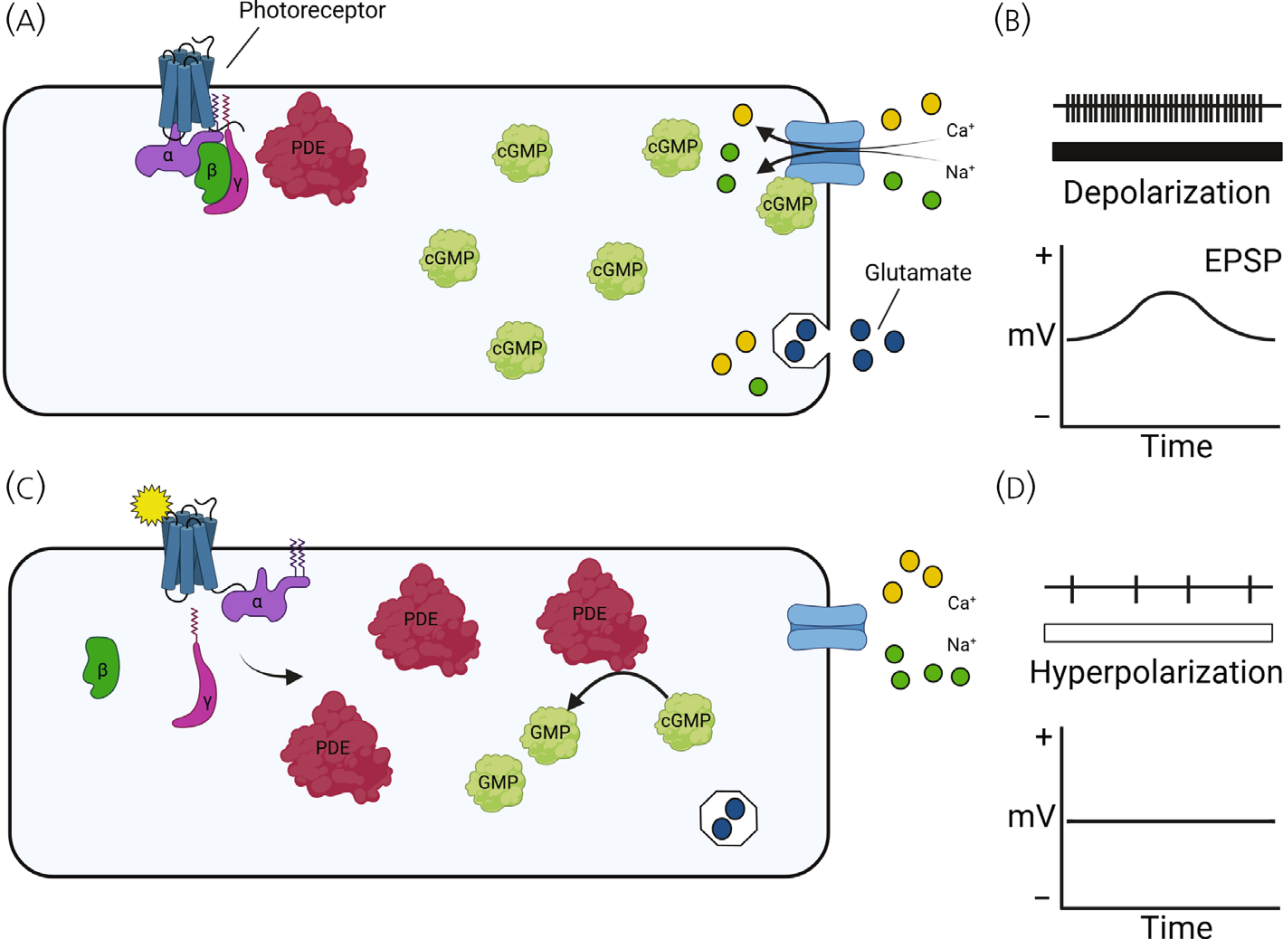
Cellular signaling transduction in photoreceptive cells. Opsin photoreceptors release the neurotransmitter glutamate. (A) In the absence of light, the G‐protein associated with opsin photoreceptors remains inactive, and there is an accumulation of cyclic guanosine monophosphate (cGMP), which actively opens ion channels to permit an influx of Sodium (Na+) and Calcium (Ca+). (B) The depolarization of the cell results in an increased release of glutamate, leading to an excitatory postsynaptic potential in post‐synaptic neurons. (C) Absorbance of a photon triggers the isomerization of cis‐retinal to all‐trans‐retinal, leading to a conformational change in opsin photoreceptors to permit the activation of G‐proteins. G‐proteins activate phosphodiesterase, which leads to hydrolysis of cGMP into the inactive GMP, resulting in closed cyclic nucleotide‐gated channels. (D) The reduction in positively charged ions results in hyperpolarization and the termination of synaptic communication. Hatched lines in (C) denote increased electrical activity in the dark (indicated by black bar). (D) The sparse electrical activity represents hyperpolarization of the cell in the presence of light (indicated by white bar).

Understanding how light is transduced by opsin cells into a signal to GnRH1 neurons is critical for describing the avian photoperiodic response. Opsin molecules are divided into ciliary (e.g., rods, VA opsin), photoisomerase (RGR), and rhabdomeric (e.g., melanopsin^4^). Initially established in 1971, and subsequently shown across ciliary opsins, is a strong hyperpolarizing graded response to light stimulation.^2^ In the absence of light, opsins are relatively inactive and result in high intracellular cyclic guanosine monophosphate (cGMP) which results in the induction of an influx of sodium and calcium ions via cyclic nucleotide‐gated cation channels to cause cellular depolarization and glutamate release (Figure 1). Light stimulation causes isomerization of 11‐cis retinal to the all‐trans form, leading to a confirmational change in the surrounding opsin protein and activation of G‐proteins. G‐proteins activate phosphodiesterase by interaction with inhibitory γ‐subunits, leading to hydrolysis of cGMP, which closes sodium ion channels and leads to hyperpolarization. The ciliary opsins are predicted to interact with G‐protein coupled receptors, and VA opsin^44^ and parapinopsin^45^ can transmit photon detection into chemical signals via transducin (G_t_ ^46^). Phototransduction by other opsins, such as neuropsin, acts via G_i_‐signaling pathways^47^ and likely inhibits adenylyl cyclase and decreases intracellular cyclic adenosine monophosphate (cAMP).^48^ Melanopsin acts via a G_q_ type G‐protein, acting via phospholipase C and inositol triphosphate leading to the opening of transient receptor potential canonical channels.^49^, ^50^, ^51^ Altogether, these patterns indicate that light detection for the avian photoperiodic response most likely involves cellular hyperpolarization in opsin‐expressing cells and consequently a reduction in glutamate release from photoreceptor cells. Therefore, for the vernal increase in photoperiod to have a stimulatory effect on GnRH1 release and subsequent gonadal growth, the neural pathway from light detection to GnRH1 synthesis and/or release must compensate for an initial inhibitory signal, such as disinhibition.

## 4 THE ROLE OF VERTEBRATE ANCIENT OPSIN FOR LIGHT DETECTION IN THE BIRD BRAIN

The avian photoperiodic response consists of three components: (1) opsin(s) that are linked to circadian clock timing, (2) the GnRH1 system, and (3) the peripheral endocrine system (e.g., gonads^52^, ^53^). Over the past two decades, the identity of the opsin(s) that link light detection to the GnRH1 system has received the most attention. The current criterion for the photoperiodic response consists of (i) neuroanatomically localized in, or directly innervating the mediobasal hypothalamus, (ii) being an opsin with a spectral maximum (*λ*_max_) of approximately 492 nm; and the spectrally filtered light captured in the hypothalamus closely matches the *λ*_max_.^54^, ^55^ Only VA opsin meets all the criteria, with mRNA and protein expression identified throughout the hypothalamus,^36^ and has an in vivo and in vitro *λ*_max_ that equals 490 nm.^37^ Recent work from our group has illustrated that transient knockdown of VA opsin mRNA and protein using short hairpin RNA increased the onset of the avian photoperiodic response, evidenced by higher GnRH1 expression during photostimulation and quicker gonadal growth.^56^

Both melanopsin^34^ and neuropsin^35^ have been proposed to link light detection to GnRH1 release in birds. However, neither fully met the criteria for phototransduction. Despite expression in the mediobasal hypothalamus,^35^, ^57^ neuropsin has a *λ*_max_ (~420 nm) that is considerably lower than the required wavelength for the avian photoperiodic response. Melanopsin has a *λ*_max_ close to the absorption spectra required for light responses,^58^ yet melanopsin expression has not been identified in the key hypothalamic brain regions.^34^ Studies that have examined the role of neuropsin for phototransduction have produced mixed results. In redheaded buntings (*Emberiza bruniceps*), the photoinduced increase in *Tshβ* in the pars tuberalis had a strong negative correlation with neuropsin.^59^ In Border canaries (*Serinus canaria*), there is also a negative correlation between *Tshβ* and neuropsin during the photoinducible phase.^57^ In the short‐photoperiod breeding Magang goose gander (*Anser anser domesticus*), there is a significant increase in neuropsin expression in response to long photoperiod while multiple transcripts involved in the activation of reproductive development including *Tshβ* and *GnRH* expression are reduced.^60^ The use of short‐hairpin RNA that specifically target neuropsin mRNA expression in both Border canaries and Japanese quail observed a significant increase in *Tshβ* expression during photostimulation.^56^, ^57^ Others have found that intracerebroventricular injections of siRNA targeting Opn5 in Japanese quail inhibit photoinduced *Tshβ* expression during the photoinducible phase.^61^ Unlike VA opsin, the transient knockdown of Opn5 was not observed to affect the GnRH1 system, nor the gonadal development during photostimulation suggest a role only during the photoinducible period of the avian photoperiodic response.

## 5 HYPOTHESES FOR PHOTOTRANSDUCTION BY VERTEBRATE ANCIENT OPSIN FOR THE AVIAN PHOTOPERIODIC RESPONSE

**FIGURE 2:**
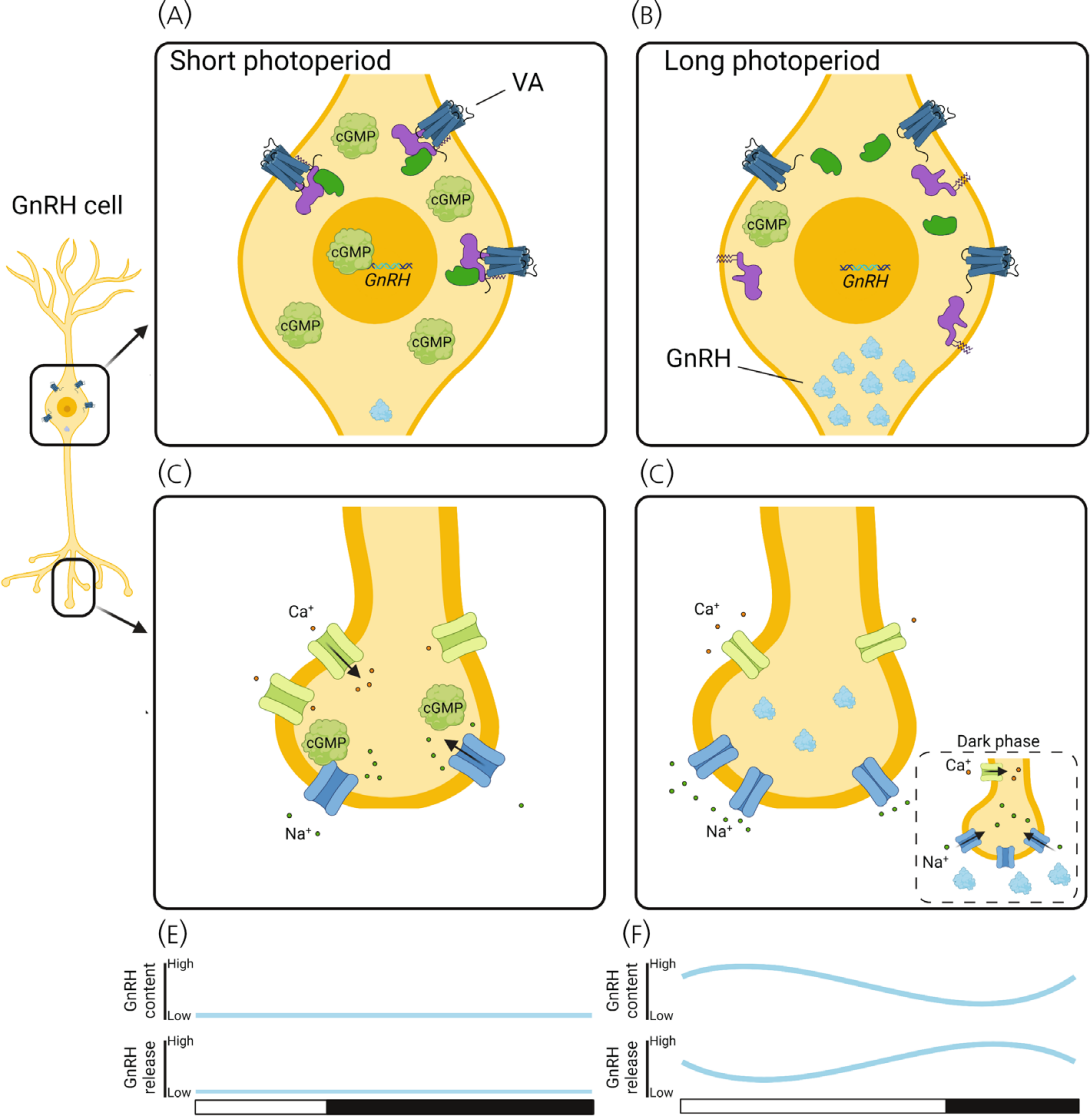
VA opsin mediated signal transduction in GnRH1 cells. In this hypothesis (A) low light detection by VA opsin in short photoperiod is predicted to result in higher intracellular cGMP and an inhibition of GnRH1 transcription. (B) Long photoperiod would be predicted to reduce intracellular cGMP and permit GnRH1 transcription. (C) Increased cGMP in presynaptic terminals results in an influx of sodium (Na^+^) and calcium (Ca^+^) ions and depolarization of the neuron. No GnRH1 peptide release occurs due to low GnRH1 transcription. (D) Increased duration of light stimulation in long photoperiod is associated with reduced intracellular cGMP, increased GnRH1 transcription, and an accumulation of GnRH1 peptide. During the dark phase, the lack of light detection by VA opsin leads to an influx of positively charged ions and the release of GnRH1 peptides. (E) Short photoperiod causes low GnRH transcription and release. (F) Long photoperiod results in high GnRH1 transcription during the light phase and increased release during the dark phase. White and black boxes indicate light and dark phases across a 24‐h period.

**FIGURE 3:**
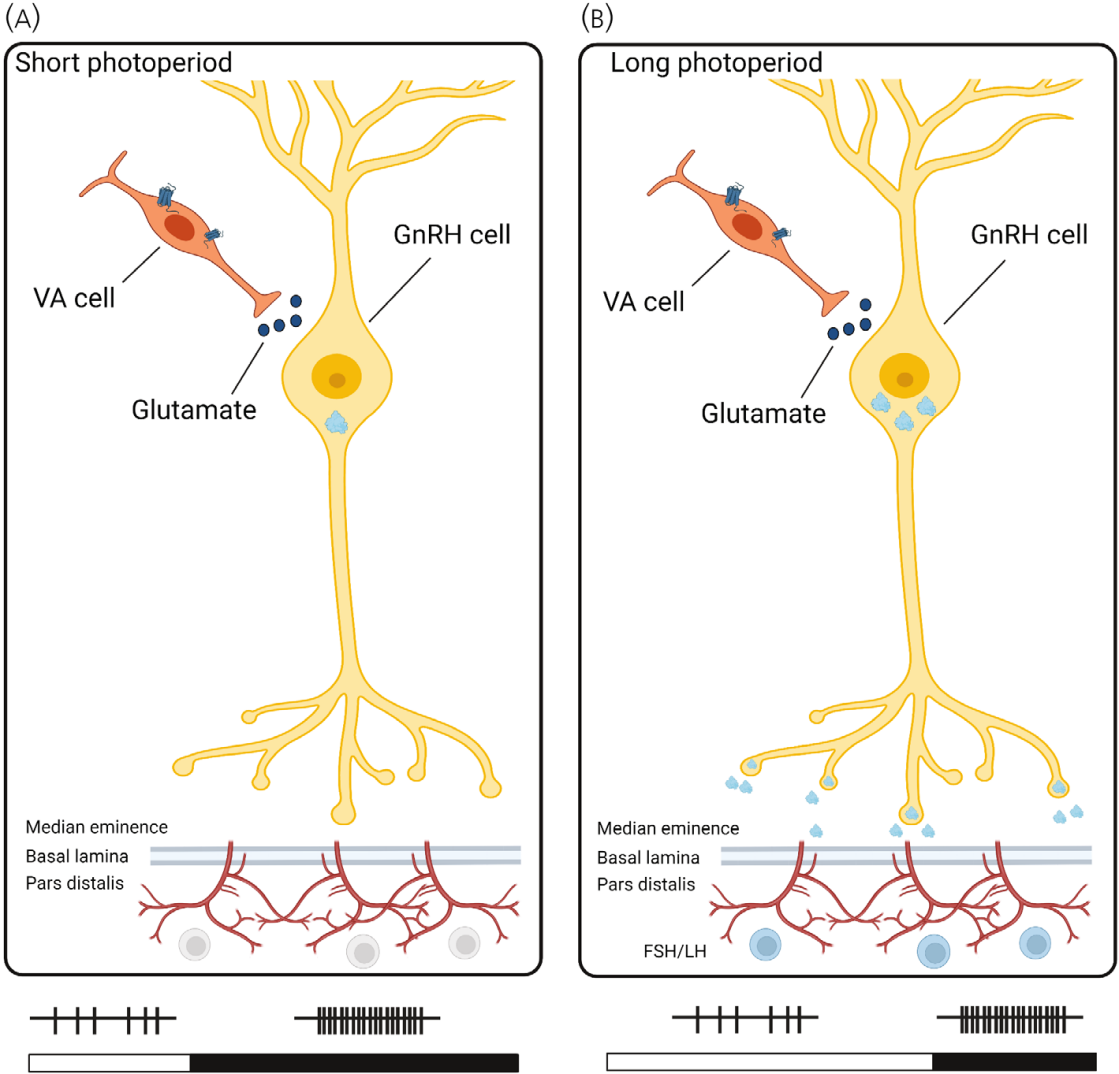
VA opsin‐mediated neuronal communication in GnRH1 cells. VA opsin cells may act as an intermediary cell between light detection and the control of GnRH release. (A) As GnRH1 transcription is inhibited in short photoperiods, there is no peptide release. (B) Under long photoperiods, the increased duration of light stimulation facilitates hyperpolarization. In the dark phase of the long photoperiod, the release of glutamate from VA opsin cells induces excitatory graded potentials and increases the probability of GnRH1 release into the hypophyseal portal system.

**FIGURE 4:**
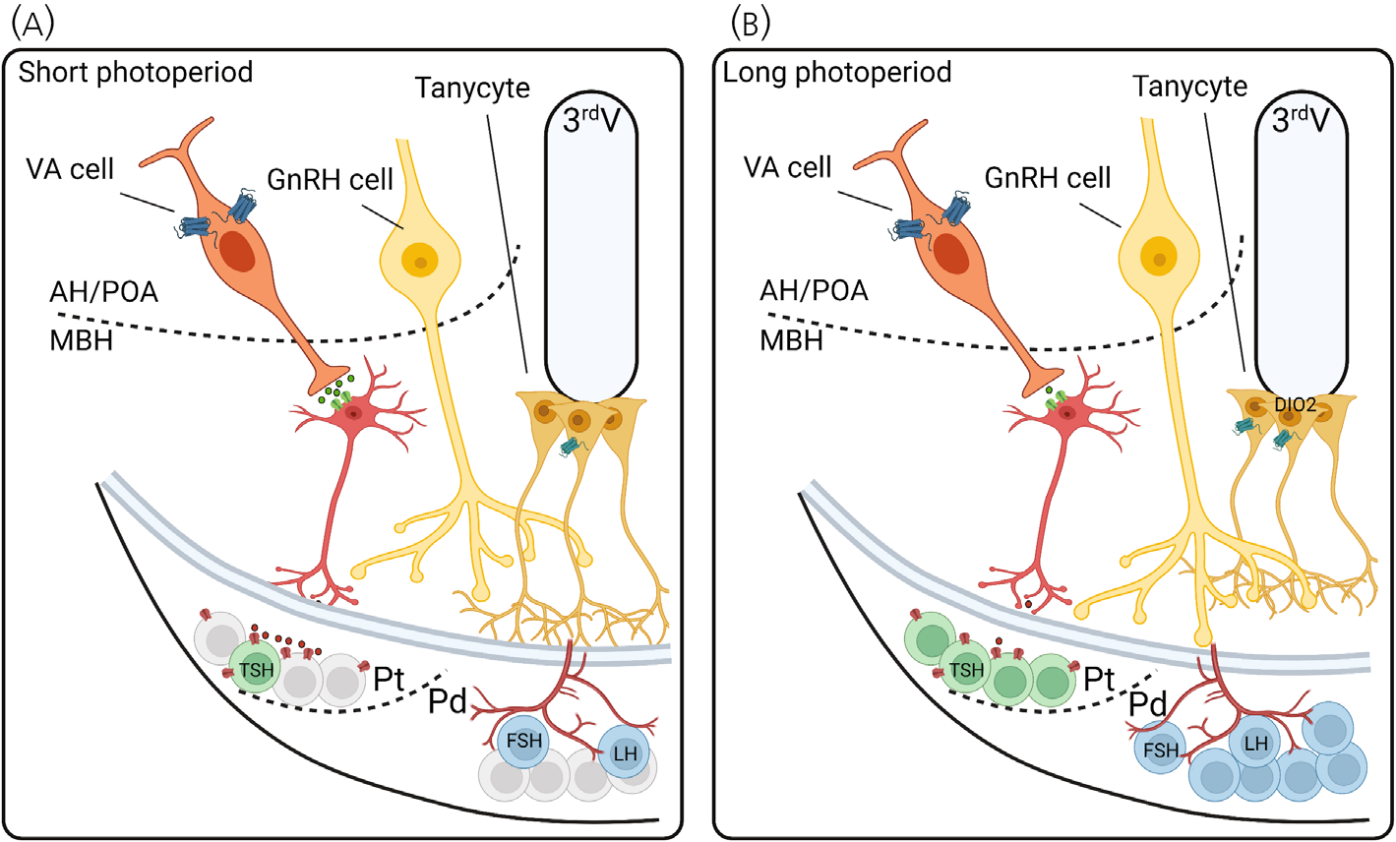
Indirect control of GnRH1 release by VA opsin. (A) VA opsin cells project into the median eminence and may act via intermediary neurons (e.g., GABA). Increased length in the dark phase causes more VA cell depolarization, and glutamate release leads to excitatory input to GABA neurons. Increased GABA activation and release into the portal vasculature inhibit thyrotropin‐stimulating hormone (TSH) cells. The absence of TSH activity causes tanycytes to extend into the median eminence and subsequently block GnRH1 release into the pituitary gland. (B) Long photoperiods cause longer durations of hyperpolarization of VA cells and a reduction in glutamate release. The decreased VA opsin input to GABA cells leads to disinhibition and the subsequent increase in TSH cell activity. TSH release binds to TSH‐receptor‐expressing cells in the tanycytes to induce retraction of terminals. GnRH1 secretion can then access the portal vasculature to stimulate the gonadotropes (luteinizing hormone (LH) and follicle‐stimulating hormone (FSH)) to release LH and FSH into the circulatory system. 3rd, third ventricle; AH, anterior hypothalamus; Pd, pars distalis; POA, preoptic area; Pt, pars tuberalis.

There are three potential scenarios in which light detection by VA opsin can regulate GnRH1 synthesis and release and subsequently gonad growth in response to photostimulation. The first hypothesis outlines how VA opsin expression co‐localized in GnRH1 cells might integrate light information to directly control GnRH1 synthesis and release (Figure 2). The second hypothesis describes how VA opsin might function as an intermediary step for light detection and directly regulate GnRH1 release (Figure 3). In the third hypothesis, we highlight how VA opsin cells might indirectly regulate GnRH1 release into the median eminence using a disinhibition step that involves an inhibitory neuron (Figure 4). This third hypothesis also outlines how VA opsin may interact with the well‐described *Tshβ* driven photoperiodic cascade, which involves the retraction of tanycytes from the median eminence, permitting access of GnRH1 neurons to the hypophyseal portal system (e.g., Yoshimura, 2013). For each hypothesis, we highlight the impact of the light–dark cycle during short‐ and long‐photoperiodic conditions. The aim of each hypothesis is to develop theoretical models in which well‐developed studies can be generated to interrogate the link between VA opsin and the avian photoperiodic response.

Hypothesis 1 Direct light detection by VA opsin in GnRH1 cells.

Immunohistochemical analyses have reported that VA opsin and GnRH1 are co‐localized in a contiguous pattern in the mediobasal hypothalamus of the domestic chicken and Japanese quail.^54^ Thus, these double‐labeled cells may contain all the components required for the avian photoperiodic response, including light detection, circadian timing, and the GnRH1 system, although circadian clock gene expression has not been quantified in these cells. As photoreceptive cells, the increased duration of darkness would result in an accumulation of cGMP^5^ (Figure 2A). Elevated cGMP is well established as an inhibitor of *Gnrh1* expression^62^ and could provide the cellular mechanism that is associated with an inhibition of GnRH1 levels during the non‐breeding season in birds.^52^ Despite the potential for increased depolarization of VA opsin cells during the dark phase under short photoperiods (Figure 2C), there is a lack of GnRH1 content to secrete, resulting in regressed gonads (Figure 2E). Conversely, under long photoperiodic conditions, the increased light exposure would reduce the level of cellular cGMP in opsin cells, facilitating *Gnrh1* transcription and GnRH1 synthesis, resulting in higher GnRH1 content during the light phase (Figure 2D) and high levels of GnRH1 are common across photoperiodic breeding birds.^17^ During the long photoperiod dark phase, increased depolarization of opsin cells would stimulate GnRH1 release, which is consistent with previous reports in hypothalamic explants from Japanese quail (Figure 2F).^63^

Hypothesis 2 Direct regulation of GnRH1 release by VA opsin cells.

Many VA opsin cells are anatomically located near GnRH1 cells in the chicken and quail mediobasal hypothalamus^36^ and could signal light cues directly to GnRH1 cells. In this hypothesis, annual changes in day length would act via VA opsin cells to regulate GnRH1 release via post‐synaptic glutamate communication (Figure 3). Under short photoperiods, opsin cells are predicted to have low hyperpolarization due to decreased light exposure and increased depolarization due to prolonged dark exposure. Due to the lack of GnRH1 in short photoperiods, especially in Japanese quail^64^ and other temperate‐zone Passeriformes^17^ the gonads are regressed. Consequently, VA opsin depolarization and the release of glutamate during the dark phase, although predicted to induce an excitatory tone on GnRH1, do not cause gonadal growth due to the low GnRH1 content (Figure 3A). In long photoperiods, the increased light exposure is predicted to facilitate hyperpolarization and an inhibitory tone during the day (Figure 3B). In Japanese quail, GnRH1 release is increased in the dark phase during long photoperiod exposure.^63^ In this scenario, the light‐dependent increase in glutamate signaling during the dark phase is predicted by an increased rate of VA opsin depolarization and GnRH1 release.

Hypothesis 3 Indirect regulation of GnRH1 by VA opsin cells.

Intermediary inhibitory connections are common across the sensory system (e.g., lateral inhibition). This hypothesis describes VA opsin‐dependent regulation of the avian photoperiodic response through the involvement of a disinhibition step that includes an inhibitory neuron (e.g., γ‐aminobutyric acid; GABA). In hypothesis 3, short photoperiods would result in a greater dark phase depolarization of VA opsin cells, leading to greater glutamate‐dependent postsynaptic signaling to an inhibitory interneuron (Figure 4A). GABA release into the anterior pituitary has long been known to inhibit secretagogue function (e.g., prolactin^65^) supporting the conjecture of neurotransmitter release into the hypophyseal portal. Increased excitatory output from VA opsin cells is predicted to stimulate more GABA release from the interneuron into the anterior pituitary gland. GABA is known to have a strong inhibitory effect on *Tshβ* expression in rat pituitary cells in culture^66^ but we do not know if this is true for birds. Long photoperiods might be predicted to reduce depolarization of VA opsin cells and consequently reduce GABA release in the anterior pituitary gland (Figure 4B). The reduction in inhibitory neurotransmitter signaling might therefore permit *Tshβ* expression. Long‐dayday‐induced *Tshβ* release, which triggers the retraction of tanycyte projections from the median eminence via a deiodinase type‐2 dependent mechanism, would permit GnRH1 release from terminals into the hypophyseal portal to stimulate gonadal growth.^30^

## 6 SUMMARY

This paper describes the neuroendocrine regulation of the avian photoperiodic response and particularly highlights that the GnRH1 system is the final common pathway across all photoperiodic bird species.^52^ The different types of opsins and the resulting phototransduction signaling cascade were outlined. The key observation for light detection by photoreceptors is in linking how hyperpolarization in response to light stimulation leads to photostimulation and gonadal growth in long photoperiod breeding species. VA opsin is the only photoreceptor that conforms to the established criteria for light detection in the bird hypothalamus. However, other opsins, including Opn4 and Opn5, are also likely involved and may act to facilitate the photoperiodic response, such as reducing noise in the light signal.^67^ We provide three theoretical hypotheses that might explain how light detection, primarily by VA opsin, can act directly in GnRH1 cells to directly regulate the release of GnRH into the hypophyseal portal system, as well as indirectly by acting via an inhibitory interneuron. Resonance experiments which examine the timing of light pulses during the photoinducible phase, despite different durations of exposure to darkness^68^ show a clear circadian function for the induction of photoperiodic genes such as deiodinase type 2 (*Dio2*) expression^69^ and the release of circulating luteinizing hormone (LH) in white‐crowned sparrows (*Zonotrichia leucophyrs gambelii* ^70^), House finch (*Haemorhous mexicanus* ^68^) and Japanese quail.^71^ These types of resonance experiments are predicted to facilitate GnRH content in the hypothalamus due to prolonged exposure to dark conditions, which is supported by evidence in both Japanese quail^64^ and European starlings.^19^, ^52^ In relation to resonance experimental design, short photoperiod induced increases in GnRH synthesis (Hypotheses 1 and 2), but the release of GnRH (Hypothesis 3) is dependent on the circadian timing of light pulses, which elevated plasma LH concentrations. It is important to emphasize that these hypotheses are models in which studies can be generated to test how light regulates VA opsin function in relation to GnRH1 synthesis and release. We propose that each hypothesis presented could potentially drive the avian photoperiodic response and they are not necessarily mutually exclusive. Although the hypotheses focus on birds that are stimulated to breed under long photoperiods, modification to each model can be extended to account for how birds like the Emu (*Dromaius novaehollandiae*) that use short photoperiods to time breeding.^72^ The primary limitation for developing a comprehensive understanding of light detection for the avian photoperiodic response is the limited number of bird species used to examine the underlying action spectrum. Overall, the scenarios described extend beyond the avian photoperiodic response, as non‐visual light detection by the brain is common across non‐mammalian vertebrates.^12^

## AUTHOR CONTRIBUTIONS

**Tyler J. Stevenson:** Conceptualization; writing – original draft; writing – review and editing; visualization; project administration. **Timothy A. Liddle:** Writing – review and editing; visualization. **Simone L. Meddle:** Writing – review and editing. **Jonathan H. Pérez:** Writing – review and editing. **Stuart N. Peirson:** Writing – review and editing. **Russell G. Foster:** Writing – review and editing; conceptualization. **Gaurav Majumdar:** Writing – review and editing.

## CONFLICT OF INTEREST STATEMENT

The authors declare no conflicts of interest.

## Data Availability

Data sharing not applicable to this article as no datasets were generated or analysed during the current study.
